# Supplementary material for: Diet and the Gut Microbiome as Determinants Modulating Metabolic Outcomes in Young Obese Adults
Source: Biomedicines. 2024 Jul 18;12(7):1601. doi: 10.3390/biomedicines12071601 (PMC11275099; doi:10.3390/biomedicines12071601)
Supplement: Supplementary file 1 [file biomedicines-12-01601-s001.zip › Table_S1.pdf]

Table S1 – Food consumption analysis using 72-hour food record (N=82)

Nutrient	Total (n=82)<br>Mean (95% CI)	Reference*
Daily energy intake, kcal per day	2984.27 (2684.36, 3284.19)	m: 2180-3800 kcal per day<br>f: 1700-3000 kcal per day
Protein intake, g per day	128.60 (116.68, 140.51)	m: 75.0-114.0 g per day<br>f: 60.0-90.0 g per day
Fat intake, g per day	132.60 (119.24, 145.96)	m: 72.0-127.0 g per day<br>f: 57.0-100.0 g per day
SFA, g per day	43.99 (39.83, 48.14)	m: 23.9-42.2 g per day<br>f: 18.9-33.3 g per day
n-6 PUFA, g per day	15.44 (11.94, 18.94)	m: 11.9-33.8 g per day<br>f: 9.4-26.7 g per day
n-3 PUFA, g per day	1.94 (1.55, 2.32)	m: 2.4-8.4 g per day<br>f: 1.9-6.7 g per day
Cholesterol intake, mg per day	506.85 (451.44, 562.26)	
Carbohydrate intake, g per day	296.15 (251.18, 341.11)	m: 301.0-551.0 g per day<br>f: 238.0-435.0 g per day
Mono / disaccharides, g per day	110.81 (98.65, 122.97)	m: 53.8-95.0 g per day<br>f: 42.5-75.0 g per day

Added sugar, g per day	22.53 (15.41, 29.65)	up to 50 g per day
Starch, g per day	66.08 (56.69, 75.47)	
Dietary fiber, g per day	27.77 (24.097, 31.44)	20-25 g per day*
Sodium intake, g per day	5004.76 (4100.62, 5908.89)	1.3 g per day
Potassium intake, g per day	3960.44 (3563.27, 4357.61)	3.5 g per day
Calcium intake, g per day	1182.49 (1062.08, 1302.895)	1.0 g per day
Beta carotene intake, mg per day	3.82 (3.07, 4.57)	5 mg per day
Vitamin B9 intake, µg per day	376.04 (327.56, 424.52)	400 µg per day
Vitamin C intake, mg per day	138.65 (116.47, 160.84)	100 mg per day
Vitamin D intake, µg per day	3.71 (2.89, 4.53)	15 µg per day

*Note: The data from Russian guidelines ref [Methodical recommendations MR 2.3.1.0253-21 «Norms of physiological needs for energy and nutrients for various groups of the population of the Russian Federation». (in Russ.)]. According to the BNF guidelines, recommended dieatary fiber intake is 30 grams per day [British Nutrition Foundation [Internet]. Nutrition.org.uk. 2021 [cited 24 May 2024]. Available from: https://www.nutrition.org.uk/; m = male; f = female. SFA = saturated fatty acid.
